# Effectiveness of a Group-Based Rehabilitation Program Combining Education with Multimodal Exercises in the Treatment of Patients with Nonspecific Chronic Low Back Pain: A Retrospective Uncontrolled Study

**DOI:** 10.3390/biology11101508

**Published:** 2022-10-14

**Authors:** Cristiano Martins, Souheil Sayegh, Antonio Faundez, François Fourchet, Hugo Bothorel

**Affiliations:** 1 Physiotherapy Department and Motion Analysis Lab, Swiss Olympic Medical Center, La Tour Hospital, 1217 Meyrin, Switzerland; 2 Department of Sports Medicine, Swiss Olympic Medical Center, La Tour Hospital, 1217 Meyrin, Switzerland; 3 Department of Orthopedic Surgery, La Tour Hospital, 1217 Meyrin, Switzerland; 4 Department of Orthopedic Surgery, Geneva University Hospitals (HUG), 1205 Geneva, Switzerland; 5 Research Department, La Tour Hospital, 1217 Meyrin, Switzerland

**Keywords:** nonspecific chronic low back pain, NSCLBP, group-based rehabilitation, physiotherapy, education, multimodal exercises, disability, high-value care, MyBack

## Abstract

**Simple Summary:**

Low back pain is a major and worldwide cause of global disability. It is not rare that an acute episode of low back pain evolves towards a chronic status without any specific cause. While many clinicians focus their treatment on low-value -care interventions (e.g., massage or electrotherapy), best clinical practice guidelines now recommend a mixed approach combining exercises and education. Furthermore, owing to the potential advantages that confer patient interactions with others (support, motivation, and program compliance), we decided to launch such an intervention program in a group setting (MyBack program) and aimed to evaluate its effectiveness through this study. Following this 8-week intervention program, approximately three-quarters of the patients reported a relevant reduction in pain intensity (78%), catastrophic thinking (78%), functional disability (74%), and fear of movement and work-related activities (74%). Only a quarter of the patients (26%) reported a relevant improvement in quality of life, probably because this outcome was already high before treatment. The MyBack program combining education with multimodal group exercises led to satisfactory clinical, functional, and psychosocial outcomes.

**Abstract:**

Currently, there is no consensus on the best rehabilitation program to perform for nonspecific chronic low back pain (NSCLBP). However, multimodal exercises, education, and group-based sessions seem to be beneficial. We, therefore, launched such a treatment program and aimed to evaluate its effectiveness in improving patient health status. We retrospectively analyzed the records of 23 NSCLB patients who followed the MyBack program at La Tour hospital from 2020 to 2022 (25 sessions, 8 weeks). Patients were evaluated before and after intervention using pain on a visual analog scale (pVAS), Roland–Morris Disability Questionnaire (RMDQ), Pain Catastrophizing Scale (PCS), Tampa Scale of Kinesiophobia (TSK), and the EuroQol-5D-3L (EQ-5D-3L). Responder rates were calculated using minimal clinically important differences. Patients reported a significant reduction (*p* < 0.05) in the pVAS (5.3 ± 1.2 vs. 3.1 ± 1.6), RMDQ (8.8 ± 3.3 vs. 4.0 ± 3.7), PCS (24.5 ± 9.4 vs. 11.7 ± 7.9) and TSK (41.5 ± 9.2 vs. 32.7 ± 7.0). The EQ-5D-3L also statistically improved (score: 0.59 ± 0.14 vs. 0.73 ± 0.07; and VAS: 54.8 ± 16.8 vs. 67.0 ± 15.2). The responder rates were 78% for the pVAS and PCS, 74% for the RMDQ and TSK, and only 26% for the EQ-5D-3L. The MyBack program combining education with multimodal group exercises led to satisfactory clinical, functional, and psychosocial outcomes.

## 1. Introduction

Low back pain (LBP) is one of the major causes of global disability in most developed countries [1,2], with 50–84% of the worldwide population affected by one or more LBP episodes in their entire life [3,4]. Low back pain can be either specific, such as secondary to trauma, malignancy, or spondyloarthropathy, or nonspecific without any known pathoanatomical cause [5]. Around 20 to 25% of LBP evolves from an acute episode towards a chronic status that is facilitated by multiple parameters such as psychosocial and physical factors as well as patient lifestyle and comorbidities [6,7]. Often defined as a symptom rather than a disease, such a condition is associated with considerable pain, functional disability, and reduced quality of life, which often affect patients in their work and daily activities [8,9].

Before starting a pharmacological or surgical treatment, best clinical practice guidelines recommend a biopsychosocial approach using exercise and education to support the self-management of nonspecific chronic low back pain (NSCLBP) [10,11]. Compared to no treatment, exercise seems to be statistically effective at reducing pain and improving functional outcomes, but with the uncertainty to be clinically relevant to patients [12]. There is, to date, no consensus on the best exercise program to perform; however, it has been reported that activities should be graded according to the patient capabilities and preferences to ensure better treatment compliance [13,14]. Although education programs alone have a minimal effect on the reduction of low back pain intensity, they were reported to have a long-term positive impact when associated with exercise in the self-management of NSCLBP, which is of particular interest for patients with persistent symptoms [15,16]. Still, many clinicians focus their treatment on low-value care interventions such as massage, ultrasounds, or electrotherapy, thereby enlarging the gap between current practice and best evidence [12,17,18].

Based on current published recommendations, an NSCLBP treatment group-based program has been recently launched at our institution combining education with multimodal exercises such as Pilates, resistance training, and aquatic exercises. Since there is little or no published data on such a treatment program, the authors aimed to evaluate and report its effectiveness in reducing pain intensity and improving functional disability, kinesiophobia, pain catastrophizing, and health-related quality of life.

## 2. Materials and Methods

### 2.1. Study Design & Participants

The authors conducted a retrospective cohort study on 29 patients with NSCLB who followed the MyBack program at La Tour hospital (Meyrin, Switzerland) from November 2020 to June 2022. Participants were included if a physician diagnosed them with NSCLBP (i.e., pain localized below the costal margin and above the inferior gluteal folds), were aged between 18 and 65 years old [13], with pain duration of at least 3 months that was not responding to a classic medication and active physiotherapy program, that referred or not to the leg [19], of intensity ranging from 4 to 10 (on a 0 to 10 scale) [20,21], and being able to read, speak and understand French. When deemed necessary, some of the patients also underwent a clinical visit with a surgeon to rule out any surgical treatment indication. Patients were, however, excluded in case of a specific cause for LBP (malignancy, vertebral fracture, infection, or inflammatory disorder) [5] or if they underwent any orthopedic surgery (spine, knee, or hip) in the previous year. Patients were also excluded if they were pregnant and if they had back surgery in the last 12 months, cardiovascular/pulmonary/metabolic/neurological and renal untreated diseases, severe osteoporosis and osteoarthritis, water phobia or other contraindication for aquatic exercise, individuals who were unable to perform exercise at a moderate-level, or other rheumatic diseases beyond NSLBP. Given this study aims at evaluating our current clinical practice based on clinical data that is routinely collected at our institution to evaluate patients’ clinical improvement, an a priori approval from our ethical committee was not required. However, all the patients included in this study gave their written informed consent for the use of their data in research projects.

### 2.2. Pre and Post-Treatment Assessment

All patients completed a questionnaire comprising patient-reported outcome measures (PROMs) before the 8-week intervention program. Outcome measures included: (i) pain intensity, measured on a Visual Analog Scale (VAS); (ii) functional disability, measured with the Roland–Morris Disability Questionnaire (RMDQ); (iii) catastrophic thinking due to low back pain, measured with the Pain Catastrophizing Scale (PCS); (iv) kinesiophobia, measured with the Tampa Scale of Kinesiophobia (TSK); and (v) health-related quality of life, measured with the EuroQol-5D-3L (EQ-5D-3L). Following the end of the intervention, patients were asked to fill up the same questionnaires and to assess their perception of overall change through the Patient Global Impression of Change (PGIC).

The pain on VAS is a simple and highly reproducible method of expressing the subjective degree of pain. The pain intensity score ranges from 0 to10, with 0 indicating no pain and 10 being the worst imaginable pain [22]. Two points on the NRS are considered as a minimal clinically important difference (MCID) in individuals with LBP [22].

The RMDQ is a 24 items questionnaire that measures the level of functional disability related to normal activities of daily living. Each item represents 1 point, and the total score varies from 0 (no disability) to 24 (extreme disability) [23], with a reported MCID of 5 points [21].

The PCS evaluates inappropriate coping strategies and catastrophic thinking about pain and injury through 13 items, with a total score ranging from 0 to 52. Higher scores indicate higher levels of catastrophizing [24]. The MCID for individuals with chronic musculoskeletal pain (CMSP) is 5.8 points [25].

The TSK is a reliable and valid tool for measuring fear of work-related activities, fear of movement, and (re)injury in patients with chronic pain. It is a 17 items questionnaire rated through a 4-point Likert scale ranging from ‘strongly disagree’ to ‘strongly agree’. The total score of the scale range from 17 to 68, where 17 means no kinesiophobia, 68 means severe kinesiophobia, and a score of ≥37 indicates there is kinesiophobia [25,26]. The MCID for CMSP patients is 4.5 points [25].

The EQ-5D-3L evaluates several important aspects of health-related quality of life, such as mobility, self-care, usual activities, pain/discomfort, and anxiety/depression, with three different levels: no problem, some problems, and severe problems. The health state is transformed into a single index score ranging from −0.59 to 1, where 1 is the best possible health state. In addition to the five dimensions, there is a VAS that assesses self-rated health on a vertical VAS, with endpoints labeled “best imaginable health state” (100) and “worst imaginable health state” (0). The EQ-5D is one of the most widely used PROMS to measure the quality of life in patients with LBP [27]. The MCID for individuals with LBP in Switzerland is 0.190 [28].

The PGIC assesses the patient’s perception of overall improvement through a 7-point scale, ranging from 1 (“no change” or “condition has got worse”) to 7 (“a great deal better” and “a considerable improvement that has made all the difference”). Patients were asked to rate the question, “Since the beginning of the treatment, how would you describe the change (if any) in your symptoms?”. Scores ≥5 were considered indicative of moderate to considerable changes in the patient’s perceived status [29,30].

### 2.3. Intervention Protocol

All participants performed a total of 25 sessions during an 8-week intervention program in a group (4 to 6 patients), 3 times per week, consisting of education and multimodal exercises such as Pilates, resistance training, and aquatic exercises. It is worth noting that group-based therapy has potential advantages owing to patient interactions with others (support, motivation, program compliance). All the interventions were provided by a trained physiotherapist with ≥3 years of experience. An overview of program details can be found in Supplementary Material Table S1.

#### 2.3.1. Education

The education program consisted of one individual session (30-min) at the beginning of the program, followed by four group sessions (90-min each) through the 8 weeks. The program content was based on general education recommendations for LBP [31], self-management, and advice to remain active [13], as well as pain neurophysiology education [32,33]. During the classes, all the concepts and information were presented and discussed using a patient-centered approach with effective and interactive communication, allowing the patient to share their pain experiences, beliefs, and fears about LBP [34,35]. The major goals were to inform the participants about LBP facts, to reconceptualize pain, to decrease fear-avoidance beliefs, to modify pain coping strategies, and to promote self-efficacy regarding the safety and benefits of movement. A “Participant Handbook’ containing the general information discussed during the sessions was given to each participant.

#### 2.3.2. Pilates

The Pilates-based mat exercises were carried out by a Pilates-certified physiotherapist and consisted of one session per week (60-min) from weeks 2 to 7, based on the protocol described by de Oliveira et al. (2019) [36]. On average, 5 to 10 exercises were performed during each session, with a maximum of 10 repetitions per exercise, divided into 3 levels of difficulty (basic, intermediate, and advanced). Exercise progression was set when participants learned how to perform 8−10 repetitions of each exercise correctly, without postural compensation and/or pain. Each exercise was selected individually, according to the objective of the sessions and the preferences of the patient. When adaptations were not possible, the exercise was substituted by another with a similar objective.

#### 2.3.3. Resistance Training

The resistance training consisted of one session per week (60-min) from weeks 2 to 7, focusing on muscle strengthening, endurance, and stabilization of the core muscle, based on the protocol described by Cortell–Tormo et al. [37]. Each session started with: (i) a 10-min warm-up of cardiovascular exercise (low to moderate intensity) on a treadmill, elliptical, bike, or row, according to patient preferences; (ii) 40-min resistance training; (iii) a 10-min cool-down with general stretching exercises for the major muscle groups. The load and intensity of each exercise were set according to the rating of perceived exertion using the OMNI resistance exercise scale, which ranges from 0 (extremely easy) to 10 (extremely hard) [38]. Free weights were used gradually during the intervention, according to the patient’s capabilities.

#### 2.3.4. Aquatic Exercises

The aquatic exercises consisted of one session per week (60-min) during all the interventions, carried out in a therapeutic pool at 32° ± 1°, as described by Abadi et al. [39]. Each session was divided into three phases: (i) 10-min warm-up, (ii) 40-min specific exercises, and (iii) 10-min cool-down, using swimming equipment, such as woggles and kickboards. The intensity was set at 60−70% of their maximum heart rate, which corresponds to a 13−17 Borg scale [40].

### 2.4. Statistical Analyses

An a priori sample size calculation to ensure fulfillment of the principal goal of the study indicated that 10 patients would be needed to detect a difference in pain on VAS of 2.0 points (MCID) with a standard deviation of 1.2 points (based on pre-treatment pain on VAS of our enrolled cohort), an alpha error probability of 0.05, a statistical power of 0.90 and an anticipated drop-out rate of 30%.

Descriptive statistics were used to summarize the data. Continuous variables were reported as mean ± standard deviation (min-max), and categorical data were reported as proportions. The normality of continuous variable distributions was assessed using the Shapiro–Wilk test. Differences between the pre-operative and post-operative values (pain on VAS, EQ-5D on VAS, EQ-5D score, TSK, PCS, and RM scales) were evaluated using either the paired student *t*-test (if Gaussian distribution) or the Wilcoxon signed-rank test (if non-Gaussian distribution). The improvements in the aforementioned outcomes were compared to their respective MCID to calculate the responder rates. The effect size of the treatment was calculated using Hedges’ g (Hedges’ g [95% Confidence Interval (CI)]) for the different studied outcomes and interpreted as follows: small (0.2 ≤ Hedges’ g < 0.5), medium (0.5 ≤ Hedges g < 0.8), large (0.8 ≤ Hedges’ g < 1.2) and very large (1.2 ≤ Hedges’ g) [41]. Statistical analyses were performed using R version 3.6.2 (R Foundation for Statistical Computing, Vienna, Austria). *p*-values < 0.05 were considered statistically significant.

## 3. Results

From the enrolled cohort of 29 patients, three (10%) refused to give their consent for the use of their data for research purposes, and three (10%) did not attend all treatment sessions. This left a final cohort of 23 patients with complete pre-and post-treatment data. This study cohort was aged 45.6 ± 11.6 years at the time of treatment and mainly comprised women (74%) and active workers (71%). Other patient characteristics can be found in Table 1.

**Figure 1:**
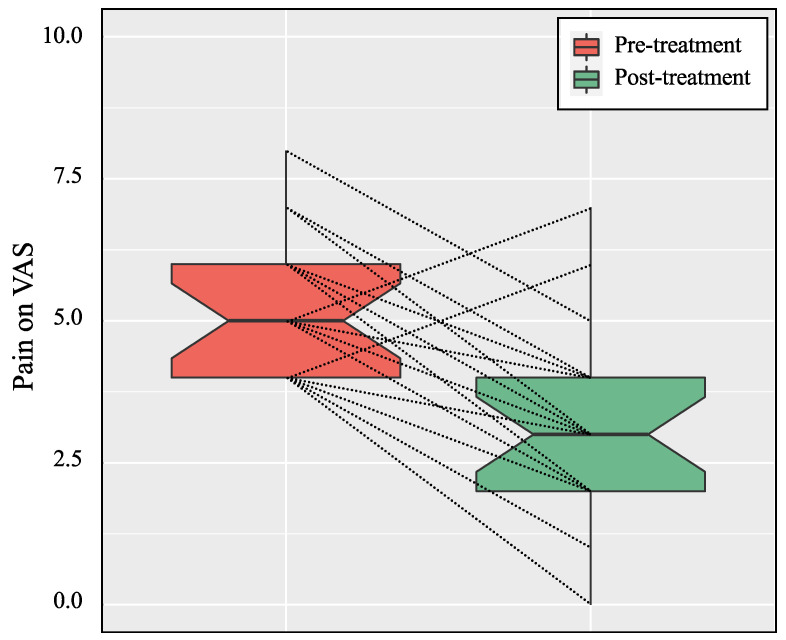
Pain on the visual analog scale (VAS) reported by the patients before and after the treatment of their nonspecific chronic low back pain (NSCLBP). The plots illustrate median values (bold lines), interquartile ranges (boxes), and 95% CIs (whiskers). Each dotted line corresponds to an individual patient change in pain on VAS between the pre- and post-treatments status.

**Figure 2:**
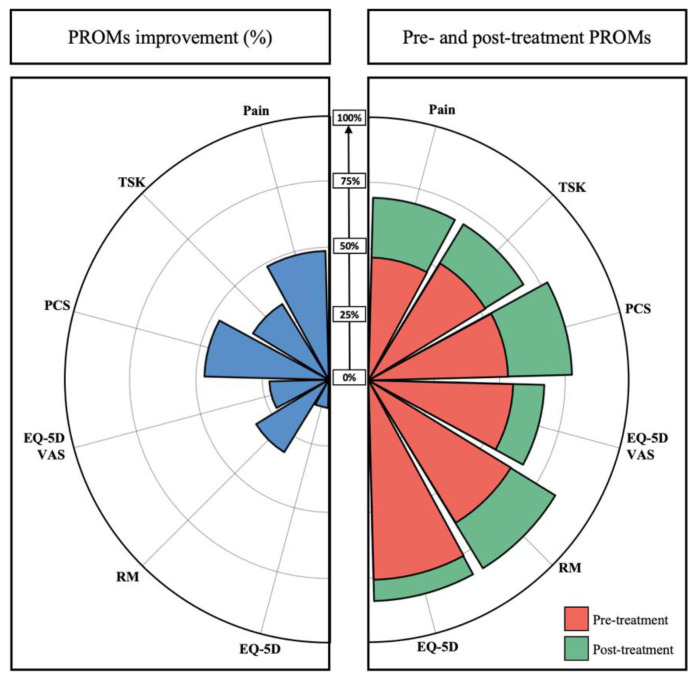
Radar chart illustrating the pre- and post-treatment patient status (right side) and the difference in % (left side). Each score was adjusted so that their minimal and maximal values were 0% and 100%, with a greater value indicating a better health status. VAS, visual analog scale; PGIC, Patients’ Global Impression of Change; TSK, Tampa Scale of Kinesiophobia; PCS, pain catastrophizing scale; RM, Roland–Morris; EQ-5D, EuroQol 5 Dimensions.

Compared to pre-treatment values, patients reported a significant reduction after treatment in pain intensity (5.3 ± 1.2 vs. 3.1 ± 1.6, *p* < 0.001) (Figure 1), RMDQ (8.8 ± 3.3 vs. 4.0 ± 3.7, *p* < 0.001), PCS (24.5 ± 9.4 vs. 11.7 ± 7.9, *p* < 0.001) and TSK (41.5 ± 9.2 vs. 32.7 ± 7.0, *p* < 0.001) (Table 2, Figure 2).

**Figure 3:**
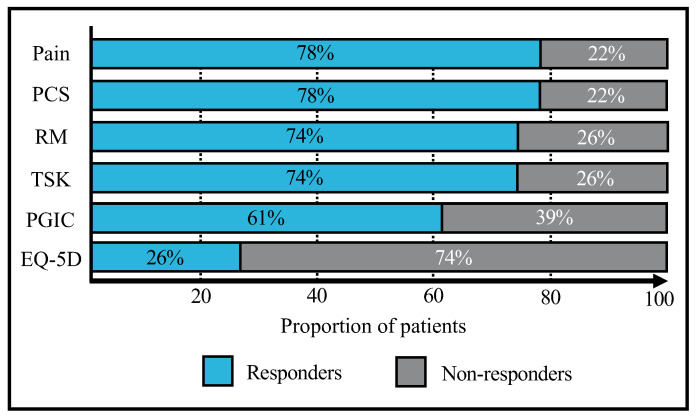
Bar chart illustrating the proportion of responders for different outcomes.

The net improvement was beyond the MCID for 78% of the patients for the pain on VAS and PCS, 74% for the RMDQ and TSK, 61% for the PGIC score but only 26% for the EQ-5D score (Table 3, Figure 3).

## 4. Discussion

Low back pain represents a major public health issue. Therefore, we implemented a treatment program combining patient education and multimodal exercises in groups at our institution and aimed at evaluating its impact on patient pain intensity, functional disability, as well as pain catastrophizing, kinesiophobia, and quality of life. The findings of the present study revealed that this intervention program has a large and positive effect on most aforementioned outcomes, with more than three-quarters of the patients reporting a relevant improvement in their pain and functional disability.

### 4.1. Improvement in Pain Intensity and Related Functional Disability

The reductions in pain intensity and functional disability observed in our patient series (2.3 and 4.9 points) compare well with those reported by Kim et al. [42] (1.9 and 3.9 points, respectively) on a comparable group of NSCLBP patients who followed an individual program combining education and lumbar stabilization exercise. Likewise, Pires et al. [43] reported a comparable pain intensity reduction of 2.3 points at 6 weeks, although only 59% of their patients achieved a change beyond the MCID. Our responder rate of 80% at 8 weeks seems, therefore, high and might be explained by (i) a high diversity of exercises, (ii) a good capacity to adapt the rehabilitation to patient needs and capacities, (iii) an appropriate balance between education and exercise sessions, and (iv) the group effect which enhances inter-patient cohesion as well as program compliance.

### 4.2. Pain-Related Psychosocial Factors

Our intervention program was effective at reducing kinesiophobia (8.9 points) and pain catastrophizing (13.2 points) with 74% and 78% of responders, respectively. Likewise, Bodez Pardo et al. [44] reported a reduction of kinesiophobia and pain catastrophizing of 8.6 points and 11.9 points, respectively, 4 weeks after the end of the treatment. This confirms that our intervention program is also beneficial for pain-related psychosocial factors. Romm et al. [45] suggested that the positive impact of group-based interventions on kinesiophobia may rely on the social observational learning of other group members suffering from a similar condition. Pain-related psychosocial factors are an important concern since individuals with NSCLBP who report low levels of physical activity tend to report higher levels of kinesiophobia and pain catastrophizing compared to patients with higher physical activity levels [46]. It is interesting to note that pain catastrophizing could be decreased by physical exercises per se, as underlined by Smeets et al. [47]. Therefore, an intervention combining exercise with education through a biopsychosocial approach seems to be an adequate option to enhance the overall positive effects of the treatment over a longer period [16,48].

### 4.3. Health-Related Quality of Life

Our results showed a large treatment effect on the EQ-5D score with an improvement that reached statistical significance. However, only 25% of the included patients were characterized as responders with a reported change above the MCID. This finding illustrates that effect sizes remain statistical results and emphasizes the importance of clinical relevance in the interpretation of PROM changes. The fact that only a quarter of our patients perceived a relevant improvement in this PROM could be explained by a relatively high health-related quality of life before treatment. Effectively, the pre-treatment quality of life reported by our patients was 20% higher (EQ-5D score, 0.60 ± 0.28 and EQ-5D on VAS, 59.0 ± 15.2) than that reported by Soer et al. [49] on a patient series suffering from a similar condition (EQ-5D score, 0.50 ± 0.28 and EQ-5D on VAS, 51.8 ± 20.9).

### 4.4. Underlying Mechanisms (Based on Scientific Literature)

Exercise has proven its effectiveness at reducing pain and disability as well as improving quality of life in individuals with NSCLBP, but the neurophysiological mechanisms are still under debate. Although the traditional main goals of exercise are to improve muscular strength and endurance, the mechanisms to reduce pain in individuals with chronic pain may not solely be due to these aspects [50]. Instead, different authors suggested that exercise decreases pain via the activation of descending inhibitory systems in the central nervous system, reducing pain sensitivity [51,52]. Likewise, the optimal type and exercise dose required to reduce pain and functional disability remain unclear. While Hayden et al. [53] found that Pilates, McKenzie therapy, and functional restoration were more effective than other types of exercise treatment in individuals with NSCLBP, other researchers claim that the type of exercise may be less important than the act of exercise itself [54,55]. This supports that the choice and content of the exercise should be individualized according to patient capacities and preferences, as well as therapist competencies, safety, and potential costs [10,52,55].

Moreover, more literature sets the importance of nonspecific (or placebo) effects and the natural course of the condition as well as psychological factors such as self-efficacy, coping strategies, and fear-avoidance beliefs to explain the effects of exercise and education on CMSP interventions [50,56,57]. Factors related to the therapist-patient relationship, such as empathy, compassion, enthusiasm, positive care, and meeting the patients’ expectations, can also influence patient beliefs about the effects of the treatment [57,58]. In a recent systematic review and meta-analysis, O’Keefe et al. [59] compared the effectiveness of different interventions programs such as physical (posture, exercise, and ergonomics), behavioral education and/or psychologically informed and combined (physical and behavioral and/or psychologically informed) but found no result differences on the reduction of pain intensity reduction and functional disability. The authors concluded that one possible reason would be that these types of intervention share the same effect mechanisms [59]. Considering the aforementioned information, it is possible that these study results were obtained via different factors, such as neurophysiological, psychological, and patient-therapist interaction in a clinical setting.

### 4.5. Limitations

This study has, however, several limitations. First, the evaluation of our institutional program is retrospective, non-comparative, and based on a small number of patients. Thus, our results might not be generalizable to other institutions, and further studies involving a community and selfcare based program (as recommended for such prevalent conditions) would be needed. Second, even though the intervention protocol’s effects grant very satisfactory short-term results, medium and long-term follow-ups have not been performed. This might be of particular importance in individuals with NSCLBP since they must deal with persistent pain and other associated symptoms over time. Furthermore, the purpose of the study was not to evaluate the results of a novel therapeutic approach (since most of its components are currently widely used and accepted), but rather to provide indications of its level of performance. Such information could, for instance, help in the design of future prospective comparative clinical trials (e.g., sample size calculation) to further evaluate the benefits of specific program components (e.g., Pilates). Finally, a selection bias must be considered. It is generally accepted that patients who are attended at different healthcare levels present differences in symptom severity. The inclusion of patients who only reported pre-treatment pain equal to or above 4 points and the single medical referral are aspects that limit the external validity of these study results.

## 5. Conclusions

The MyBack rehabilitation program combining education with multimodal exercises in a group led to satisfactory results with a relevant reduction of pain intensity and functional disability for more than 74% of our patients suffering from NSCLBP. Likewise, fear of movement and catastrophic thinking related to pain decreased to the same extent, illustrating the global benefits of such a rehabilitation program on clinical, functional, and psychosocial patient status.

## Figures and Tables

**Table 1:** Characteristics of the original cohort.

	Original Cohort (*n* = 23 Patients)		
	Mean	±SD	(Range)
	*n*	(%)	
Patient characteristics			
Sex			
Women	17	(73.9%)	
Men	6	(26.1%)	
Professionally active	17	(70.8%)	
Age	45.6	±11.6	(29.0–64.0)
BMI	23.7	±3.4	(18.9–30.1)
Weight (kg)	67.8	±13.3	(50.0–102.0)
Height (m)	1.69	±0.10	(1.55–1.85)

BMI, Body Mass Index; SD, Standard deviation.

**Table 2:** Pre- and post-treatment PROMs comparison.

	Pre-Treatment (*n* = 23 Patients)			Post-Treatment (*n* = 23 Patients)			
	Mean	±SD	(Range)	Mean	±SD	(Range)	*p*-Value
Pain on VAS	5.3	±1.2	(4.0–8.0)	3.1	±1.6	(0.0–7.0)	<0.001 **
Roland Morris	8.8	±3.3	(2.0–17.0)	4.0	±3.7	(0.0–13.0)	<0.001 **
PCS	24.5	±9.4	(3.0–40.0)	11.7	±7.9	(2.0–26.0)	<0.001 *
TSK	41.5	±9.2	(27.0–62.0)	32.7	±7.0	(20.0–47.0)	<0.001 *
EQ-5D-3L score	0.59	±0.14	(0.31–0.78)	0.73	±0.07	(0.57–0.85)	<0.001 **
EQ-5D-3L on VAS	54.8	±16.8	(20.0–90.0)	67.0	±15.2	(40.0–90.0)	0.004 *

VAS, visual analog scale; PCS, Pain Catastrophizing Scale; TSK, Tampa Scale of Kinesiophobia; BMI, Body Mass Index; SD, Standard deviation; PROM, patient-reported outcome measurement. * Pre- to post-treatment difference evaluated using a paired Student *t*-test. ** Pre- to post-treatment difference evaluated using a Wilcoxon signed rank test.

**Table 3:** Responder rate according to MCID values.

	Final Cohort (*n* = 23 Patients)				
	Net Improvement			Responders *	
	Mean	±SD	(Range)	N	(%)
Pain on VAS (MCID = 2.0 pts)	2.3	±1.7	(−2.0–4.0)	18	(78.3%)
Roland Morris (MCID = 5.0 pts)	4.9	±2.9	(0.0–10.0)	17	(73.9%)
PCS (MCID = 5.8 pts)	12.9	±9.8	(−7.0–36.0)	18	(78.3%)
TSK (MCID = 4.5 pts)	8.9	±6.8	(−2.0–23.0)	17	(73.9%)
EQ-5D-3L score (MCID = 0.190 pts)	0.13	±0.14	(0.00–0.46)	6	(26.1%)
PGIC (MCID = 5 pts)	4.4	±1.6	(1.0–7.0)	14	(60.9%)

VAS, visual analog scale; PCS, Pain Catastrophizing Scale; TSK, Tampa Scale of Kinesiophobia; BMI, Body Mass Index; SD, Standard deviation; PROM, patient-reported outcome measurement; Patients’ Global Impression of Change (PGIC) scale; MCID, minimal clinically important difference. * Responders indicate the number of patients who improved their PROM beyond the MCID.

## Data Availability

Data supporting reported results can be requested from the corresponding author (hugo.bothorel@latour.ch).

## Supplementary Materials

The following supporting information can be downloaded at: https://www.mdpi.com/article/10.3390/biology11101508/s1, Table S1: Table describing in detail the 8-week intervention program.
