# Supplementary material for: Effectiveness of a Group-Based Rehabilitation Program Combining Education with Multimodal Exercises in the Treatment of Patients with Nonspecific Chronic Low Back Pain: A Retrospective Uncontrolled Study
Source: Biology (Basel). 2022 Oct 14;11(10):1508. doi: 10.3390/biology11101508 (PMC9598691; doi:10.3390/biology11101508)
Supplement: Supplementary file 1 [file biology-11-01508-s001.zip › biology-1958225-supplementary.pdf]

Table S1: Description of the 8-week intervention program

**Education (5 sessions)**	**Individual session (30min) - Individual initial assessment**<br><br>**Topic 1**: introducing the program to the patient.<br>**Topic 2**: understanding the participants' cognitions, beliefs and attitudes towards pain and the behaviours adopted in their daily life activities.<br>**Topic 3**: establishing SMART goals for the program.	**Group session 1 (90min) - Meeting the group**<br><br>**Topic 1**: listening each patient's LBP history and promoting dialogue between the group.<br>**Topic 2**: providing general information about myths and facts in LBP while challenging the patient's beliefs about LBP.	**Group session 2 (90min) - Understanding Pain Neurophysiology**<br><br>**Topic 1**: increasing the knlowedge regarding pain: from acute to chronic MSK pain.<br>**Topic 2**: understanding the role of the brain, as well as the psychosocial and behavioral factors on the maintance of chronic pain.<br>**Topic 3**: managing pain flare-ups with graded physical activity to reduce symptoms.	**Group session 3 (90min) - Posture, ergonomics and loads**<br><br>**Topic 1**: challenging the behaviours about pain, posture and carying objects.<br>**Topic 2**: practical simulation: posture and load management<br>**Topic 3:** self-management exercises during prolonged sitting position.	**Group session 4 (90min) - Final assessment**<br><br>**Topic 1**: reviewing the principal information about LBP and chronic MSK pain.<br>**Topic 2**: assessing each participant goals and general participation in the program.
**Pilates based-mat exercises (6 sessions of 60min)**	**Basic**		**Intermediate**	**Advanced**	
	**Spine exercises:** bridge; rolling back down/up (knees flexed and rolling forward); hamstring stretch; swan front; tower (V position with elastic forward and upward); mermaid (both hands on swissball).		Bridge variant (swissbal); rolling back down/up (knees extended and rolling forward); hamstring stretch (crossed legs); swan (taking off the arms); long spine (V position with elastic forward and upward); mermaid (one hands on swissball).	Single-leg bridge; rolling up (rolling back and forward); hamstring stretch (swiss ball); swan (taking off arms and legs); jackknife; side arm sit variant.	
	**Lower Limb Exercises** footwork double legato (four supports rising one leg); side kicks up and down; side kicks inner-thigh lifts; fire hydrant; horse.		Footwork double legato (four supports with mild elastic band); side kicks up and down (mild weight); side kicks inner-thigh lifts (mild weight); fire hydrant (mild elastic); horse (mild elastic).	Footwork double legato (four supports with heavy elastic band); side kicks up and down (heavy weight); side kicks inner-thigh lifts (heavy weight); fire hydrant (heavy elastic); horse (heavy elastic).	
	**Upper Limb Exercises**: arms biceps (with elastic); arms pull up and down; arms triceps (mild elastic).		Arms biceps (flexed legs with elastic); arms pulling (mild elastic); arms triceps (mild elastic without feet support).	Arms biceps (standing with elastic); arms pulling variant (heavy elastic); arms triceps (heavy elastic without feet support).	
	**Abdominal Exercises**: teaser; the hundred (pumpings arms and stretching legs); prone bridge with knees and feet supported.		The hundred variant (with feet on the air); the hundred (raising the swissball); prone bridge with knees supported.	Exercise with swissball; the hundred variant (side); prone bridge.	
**Resistance training (6 sessions of 60min)**	**Warm-up (10min):** Cardiovascular exercise (treadmill, elliptical, bike or row), low to moderate intensity.				
	**Stage 1: 2 sessions** (OMNI: 4)<br>Isolated movements and isometric control, with controlled respiration in weight bearing with neutral lordosis.<br>11 exercices circuit, 20 reps/No rest between exercices, 1-2 sets<br>**Exercises:** specific isometric co-contraction of tranversus abdominus.		**Stage 2: 2 sessions** (OMNI: 6-7)<br>Isometric and analytic control: 9 exercices circuit, 15 reps/set, 30sec active rest, 2-3 sets.<br>**Exercises:** abdominal crunch, back extension, side plank, elbow bridge, squat, lunges, seat pull and row.	**Stage 3: 2 sessions** (OMNI: 8-9)<br>Isometric, analytic and integrated chain control<br>11 exercices, 12 reps/set, 30 sec active rest, 3 sets.<br>**Exercises**: Single leg Deadlift. Anterior Reach. Lunges. Stand row. Pull squat. Stand push.	
	**Cool-down (10min):** General stretching exercises for the major muscle groups, holding 10-30 sec.				
**Aquatic exercise (8 sessions of 60min)**	**Warm-up (10min):** 1- General mobility exercise, increasing range of motion; 2- Static walk.				
	**Main exercises (30min):** 1 - Walter walking / jogging (forward and backward); 2 - forward and side lundges; 3 - Supine postion with hip and knee bent; 4- Supine position+cycling; 5- Shoulder transvers (abduction, adduction, flexion and extension); 6 - Upper body mobilisation (with kickboard); 7 - Wall push-up; 8 - Deep water cycling.				
	**Cool-down (10min):** 1 - General stretching of the major muscle grounps, holding 10-20 sec; 2 - Deep breathing technique and relaxation; 3 - Sel-care free water-activity.				
